# Effects of Seedling Grade on Biomass Accumulation and Allometric Growth Across Developmental Stages in *Pinus yunnanensis* Franch. Seedlings

**DOI:** 10.3390/biology15191692

**Published:** 2026-09-23

**Authors:** Pengrui Wang, Honglin Wang, Zhuangyue Lu, Xiangyang Kang, Yulan Xu, Nianhui Cai, Boning Yang

**Affiliations:** 1 The Key Laboratory of Forest Resources Conservation and Utilization in the Southwest Mountains of China, Ministry of Education, Southwest Forestry University, Kunming 650224, China; 18387876686@163.com (P.W.); whonglin0913@163.com (H.W.); luzhuangyue@swfu.edu.cn (Z.L.); xuyulan@swfu.edu.cn (Y.X.); 2 The Construction Program of Yunnan Provincial Key Laboratory for Conservation and Utilization of In-Forest Resource, Southwest Forestry University, Kunming 650224, China; 3 State Key Laboratory of Tree Genetics and Breeding, National Engineering Research Center of Tree Breeding and Ecological Restoration, College of Biological Sciences and Biotechnology, Beijing Forestry University, Beijing 100083, China; kangxy@bjfu.edu.cn

**Keywords:** *Pinus yunnanensis* Franch., seedling grading, biomass accumulation, growth pattern

## Abstract

*Pinus yunnanensis* Franch. holds significant ecological value as a native conifer endemic to the southwestern uplands of China. The annual *P. yunnanensis* seedlings were categorized into three quality classes: Grade I, Grade II, and Grade III in accordance with the national specifications for seedling quality. We measured root, stem, needle, aboveground, and individual biomass at six stages from 240 to 690 days. We used the standardized major axis method to analyze allometric relationships among organs and with total biomass. Our results showed that Grade I seedlings had more stable allometric coordination over time. The lower grades showed more fluctuations and weaker coordination. Needles dominated biomass throughout the development period, roots grew faster in later stages, and stems grew slower but increased their share. This showed seedlings invest first in needles for carbon gain and then gradually build support and uptake structures. The biomass allocation and growth modes matured between 510 and 690 days. Biomass differed among seedling grades. Grade I seedlings show better growth stability and resource-use efficiency. They are suitable for nursery cultivation and afforestation. This study lays the groundwork for upgrading quality standards and perfecting silvicultural techniques. It also helps design precise tending measures to raise survival rates and early productivity. This work has practical value for plantation establishment, ecological restoration, and timber development in Southwest China.

## Simple Summary

*Pinus yunnanensis* Franch. is an important native tree in Southwest China. We categorized the annual seedlings into Grade I, Grade II, and Grade III based on national criteria. We measured root, stem, needle, aboveground, and individual biomass at six different developmental stages from 240 to 690 days. We also analyzed allometric relationships among organs. The needles always dominated biomass accumulation. The organ allocation strategies and growth patterns became established between 510 and 690 days. Biomass accumulation and resource use strategies differed significantly among seedling grades. Grade I seedlings grew steadily and exhibited significant advantages in biomass accumulation, with more stable allometric relationships and stronger organ coordination. They are suitable for nursery cultivation and afforestation. This study provides a theoretical basis for precise tending measures. These measures can improve survival rates and early productivity. The findings also have practical value for plantation establishment, ecological restoration, and timber industry development in Southwest China.

## 1. Introduction

Biomass is a fundamental indicator reflecting plant growth status, resource utilization efficiency, and environmental adaptation [1]. As the material basis for plant survival and development, biomass accumulation and allocation directly determine individual plant fitness, population stability, and ecosystem function [2,3]. Biomass allocation describes how photosynthetic products are distributed among roots, stems, needles, and other organs during plant growth [4]. Furthermore, allocation strategies develop through long-term environmental adaptation and evolutionary selection [5]. Consequently, a reasonable biomass allocation pattern helps plants maximize resource acquisition and maintain steady growth in natural habitats [6]. Extensive scientific research has examined biomass allocation. Evidence from previous studies indicates that biomass allocation is critically important for seedling morphogenesis and vigor [7]. However, systematic analyses of dynamic biomass accumulation and allocation patterns across developmental stages remain limited. Therefore, such analyses are theoretically important for exploring plant growth strategies and environmental adaptation mechanisms [8,9].

To address this gap, the allometric approach is a fundamental tool for quantifying growth correlations between aboveground and belowground components [10]. This theory quantitatively describes the relative growth rates and correlations between roots, stems, needles, and individual plants, which can directly reflect plant resource allocation strategies and growth balance [5,11]. Previous studies have confirmed that stable allometric growth relationships generally exist among plant organs, and these relationships are rarely affected by short-term environmental changes. Moreover, this stability helps clarify plants’ inherent growth rules [12,13]. In particular, for woody plants, especially coniferous species, biomass allocation and allometric growth characteristics at the seedling stage are closely related to afforestation survival rate, early growth vigor, and stand productivity [14,15]. Therefore, exploring the allometric relationships among seedling organs and biomass allocation has become a key research focus. This helps reveal tree growth and development mechanisms.

Beyond allometric relationships, the external morphology and growth stages of seedlings at different grades exhibit discernible differential characteristics [16]. After obtaining the mean values and deviations of each trait, a clustering strategy was adopted, in conjunction with silvicultural practice standards, to classify the test seedlings into Grade I, Grade II, and Grade III [17]. Grade I and II seedlings are good for planting in forests [18]. In contrast, Grade III seedlings are not yet ready, so they need longer care before use [18]. Notably, researchers tracked *Pinus elliottii* and *Pinus taeda* seedlings for 13 years [19]. They found that the seedling performance followed a clear grade-dependent hierarchy, with Grade I outperforming Grade II, which in turn outperformed Grade III in both survival and growth [19]. Moreover, the other study also cited Wakeley’s experiment and further proved that Grade I seedlings had a long-term advantage [19]. Collectively, these findings demonstrate that seedling quality is very important for forest planting. Therefore, seedling quality directly is associated with survival after planting and subsequent forest growth.

In this context, *Pinus yunnanensis* Franch. is a unique coniferous species native to southwest China, with strong adaptability, fast growth rate, and prominent ecological and economic values [20,21]. Combining the market value approach and the optimal forest carbon sink pricing method, among the tree species, *P. yunnanensis* contributed the most, accounting for 87.51% of the economic value of carbon storage [22]. This species occurs across a wide range in Yunnan, Sichuan, and Guizhou and provides critical ecosystem services, including water and soil retention, carbon fixation, oxygen release, wood production, and oleoresin yield [23,24]. Furthermore, *P. yunnanensis* is employed in the production of high-value solid wood commodities, including furniture and paper, while its turpentine oil finds extensive application in both pesticides and biofuel production [25]. As a major afforestation and timber tree species, *P. yunnanensis* has been widely studied for its growth characteristics and biomass production capacity [26]. At present, studies on *P. yunnanensis* mainly focus on community structure, nutrient cycling, stress resistance, and cultivation techniques [27,28]. More recently, research has increasingly focused on biomass accumulation and allometric growth patterns. For example, recent work has examined how shading intensity modulates biomass allocation and allometric growth responses in decapitated seedlings [29], and how decapitation alters biomass allocation across a gradient of shading conditions [30]. In addition, recent studies have clarified the allometric growth patterns of different seedling grades at various developmental stages [31] and explored the interdependence of biomass allocation and allometric growth across seedling quality classes [9]. However, systematic research on this topic is still unclear. The coupling between biomass allocation and allometric growth has also been explored in grade-classified seedlings. The scope should integrate biomass accrual, organ-level distribution, and size-dependent growth across seedling grades throughout the ontogeny of *P. yunnanensis* under field settings. We measured organ biomass at different developmental stages. We evaluated seedling quality and analyzed allometric growth among grades. We also proposed two hypotheses. First, grade and biomass allocation will show a clear positive correlation over time across different stages. Second, allometric growth patterns will differ among grades. Grade I seedlings will have more stable allometric relationships and better organ coordination.

However, this study used a different sampling cohort from two related *P. yunnanensis* studies. One related study sampled 1448 annual seedlings and analyzed morphological data across developmental stages but lacked biomass data [31], whereas another related study sampled 185 two-year-old seedlings and analyzed biomass data but lacked temporal dynamics [9]. In contrast, this study used 162 annual seedlings and integrated biomass data across six developmental stages from 240 to 690 days. The new contribution of this study is therefore a temporal biomass dataset that enables quantification of how seedling grade affects biomass accumulation and size-dependent growth across developmental stages.

To fill this gap, this study aimed to explore how different seedling grades affect component biomass accrual and size-dependent growth in *P. yunnanensis* seedlings. We wanted to reveal the growth strategy differences among grades at different developmental stages. Using longitudinal tracking of six stages and three grades with allometric modeling, this study found that *P. yunnanensis* seedlings complete the transition from collaboration to division of labor from 510 to 690 days. Specifically, Grade I seedlings excel due to stable organ coordination, while inferior seedlings face a persistent threshold effect. Notably, needle dominance shifts from isometric coordination to strategic tilting in the middle and late stages, and low-grade seedlings show compensatory resource-use constraints. Importantly, this study provides unique evidence on early development in subtropical conifers. Meanwhile, longitudinal data reveal, for the first time, a stage-wise dynamic shift in needle-leaf dominance, from early isometric coordination to mid-late strategic reallocation, breaking through the limitations of previous static comparisons. This research provided basic data for understanding resource allocation strategies in subtropical conifer species during ontogeny. In practice, our findings can guide seedling grading, selection of superior seedlings, and choice of planting materials. They can also help improve survival rates and early stand productivity.

## 2. Materials and Methods

### 2.1. Site Characteristics and Experimental Layout

The experimental site was located in the greenhouse of Southwest Forestry University in Kunming, Yunnan Province (25°04′00″ N, 102°45′41″ E) [31]. The site sits at an altitude of about 1945 m and belongs to the subtropical plateau monsoon climate zone. The local annual mean temperature is 14.7 °C, with an extreme maximum of 32.5 °C and an extreme minimum of −9 °C. The annual average relative humidity is 68.2%, and annual precipitation ranges from 700 mm to 1100 mm, mostly concentrated between May and October. The area enjoys 2445.6 h of annual sunshine, with a sunshine percentage of 56%. The annual total solar radiation averages 543.36 kJ/cm^2^, and annual evaporation is 1856.4 mm. The local soil is a typical acidic, low-phosphorus red soil.

### 2.2. Geographic Origin of Seed Materials

First, experimental seeds were collected from the Yunnan Pine clonal seed orchard in Midu County, Yunnan Province. Well-grown mother trees were selected, and fully mature current-year cones were gathered from the superior seed base of *P. yunnanensis*. After being labeled, the cones were transferred to the laboratory, where they were arranged in a single layer for natural air-drying. Then, full and uniformly sized seeds were screened for subsequent use. Subsequently, the disinfection procedure was as follows: seeds were soaked in 0.5% potassium permanganate for 1 h, rinsed with tap water, and immersed in warm water for 24 h.

Meanwhile, we disinfected the cultivation soil and seedling trays with the same potassium permanganate solution. After sowing, the seedbed was covered with pine needles and a film shed to conserve moisture. Greenhouse management included regular watering, weeding, and other necessary measures according to the prevailing weather conditions and seedling developmental stages. After germination, we transplanted 100 days seedlings into seedling pots. We mixed the culture substrate with humus soil and red soil at a 3:1 volume ratio. We selected seedlings with consistent growth for follow-up tests. During the cultivation period, we implemented uniform management: weeding was carried out every two weeks, and watering was performed every 3–5 days to keep the substrate sufficiently moist. All experimental *P. yunnanensis* seedlings were grown under identical nursery conditions, with a completely randomized arrangement, and all abiotic factors were held constant.

### 2.3. Seedling Grading

We raised all *P. yunnanensis* seedlings under identical environmental settings and used a completely randomized arrangement to minimize positional effects. They also needed to match production practices and be practical and easy to use. Studies show that using seedling height as a grading criterion is intuitive and highly operable, with no major difference compared to ground-line diameter analysis [9,31,32]. For this reason, we used seedling height as the only morphological indicator for grading. The experiment was conducted on over 1900 seedlings, whose initial morphometric data provided the foundation for all subsequent quality grading. In accordance with national and regional quality specifications, seedling classification was performed using a height-based criterion defined as the mean height ± half of its standard deviation, with the specific formula presented below [33] (Figure S1).

G = ω ± 1/2 σ(1)

In this formula, G represents the grading standard, ω represents the average seedling height, and σ refers to the standard deviation of the height measurements for the seedling population. Seedlings were divided into three grades in accordance with national and local seedling quality standards (LY/T 1950-2011) [34].

The size threshold for Grade I seedlings was derived from the standard equation presented below:

I ≥ ω ± 1/2 σ(2)

The size threshold for Grade II seedlings was derived from the standard equation presented below:

ω − 1/2σ ≤ II < ω + 1/2σ(3)

And the size threshold for Grade III seedlings was derived from the standard equation presented below:

III < ω − 1/2σ(4)

Following grading, seedlings of each grade were reared under uniform growing conditions before biomass assessment.

### 2.4. Biomass Measurement

After seedling grading of *P. yunnanensis*, nine seedlings with consistent growth vigor were randomly selected from each grade for biomass determination. Each grade had 9 seedlings, representing 9 biological replicates. There were 3 seedling grades in total, giving 27 seedlings per sampling date. Over 6 sampling dates, a total of 162 seedlings were sampled. This experiment did not include technical replicates. We sampled and measured the seedlings at six key stages: 240 d, 330 d, 420 d, 510 d, 600 d, and 690 d, with one measurement at each stage. We harvested the seedlings as whole plants, taking care not to injure any tissue during excavation. We partitioned each sampled seedling into three fractions: roots, stems, and needles. We weighed each organ fresh on a 0.001 g precision balance and recorded the weights. We transferred each plant sample to a labeled paper envelope. We oven-dried samples at 105 °C for 30 min and then dried them at 80 °C to a constant weight [35,36]. We determined and recorded the dry mass of each organ, which we used as the organ biomass. Aboveground biomass was calculated as the sum of stem and needle biomass, while individual plant biomass referred to the total biomass of root, stem, and needle.

### 2.5. Data Analysis

We used Excel 2021 (Microsoft Corp., Redmond, WA, USA) to organize and sort the raw data. We then used SPSS Statistics 27.0 (IBM Corp., Armonk, NY, USA) for one-way and two-way analysis of variance and Duncan’s multiple range test.

Allometric growth describes a non-linear quantitative relationship [37]. This relationship is often shown as Formula (5):

*y* = *a x ^b^*, (5)

In this formula, one variable grows at a rate that is proportional to another variable’s growth rate. Then we get a linear relationship shown as Formula (6):

*log y* = *b log x* + *log a* (6)

In this formula, *y* refers to the total biomass of individual seedlings, and *x* represents the biomass of different components, including root, stem, needle, and aboveground part. The intercept of the organ–biomass relationship was denoted as *log a*, and the slope as *b*, which was also interpreted as the allometric exponent for biomass distribution among grades. Isometric growth was inferred when the confidence interval of *b* included 1, and allometric growth was inferred when it did not.

We used the SMATR package in R software (version 3.4-8) and applied the standardized major axis estimation method [38]. We obtained the parameter estimates. We performed multiple comparisons. These comparisons tested whether the fitted axes were equal. All data are shown as mean ± standard error. All figures were drawn using Origin Pro 2025 (OriginLab Corp., Northampton, MA, USA).

## 3. Results

### 3.1. Biomass Accumulation and Variation Characteristics of Individual Seedlings for Different Seedling Grades at Different Developmental Stages

The two-way ANOVA results in Table 1 showed that individual biomass differed significantly among grades, developmental stages, and their interaction. The significant interaction indicated that biomass trajectories across stages differed significantly among grades; therefore, differences among grades depended on developmental stage, and interpreting only main effects could obscure biological patterns. In summary, all factors showed very large effect sizes. Developmental stage showed the largest effect size, with a partial eta-squared value of 0.857, making it the dominant factor associated with accumulation. Seedling grade followed, with a partial eta-squared value of 0.671, and their interaction had a partial eta-squared value of 0.509. This indicates that the magnitude of differences among grades changed across developmental stages and that their joint contribution cannot be ignored.

Accordingly, based on the significant interaction, we conducted further analysis. As shown in Figure 1, the individual biomass among grades at the six developmental stages is presented. During the seedling cultivation period from 240 to 690 days, individual biomass increased monotonically with grade, with persistent biomass accumulation throughout the grading sequence. The three seedling grades showed generally consistent growth patterns when compared at different growth stages within the same grade. For Grade I seedlings, biomass was lowest at 240 days and differed significantly from other seedling stages. No significant difference and similar biomass levels were observed between 330 days and 420 days. Biomass increased significantly at 510 days and 600 days and reached its maximum at 690 days, which was significantly higher than at all previous seedling stages. For Grade II and Grade III seedlings, biomass did not differ significantly at 240, 330, and 420 days. Significant differences in biomass appeared at 510 days compared with the early growth stage. Biomass further improved, with significant differences at 600 days, and reached a relatively high level at 690 days, which was significantly different from all previous seedling ages. Under the same developmental stages, individual biomass generally decreased from Grade I to Grade II and Grade III. At 240 and 420 days, Grade I seedlings exhibited a statistically significant biomass superiority over Grade II and Grade III seedlings. At four time points from 330 days to 690 days, Grade I seedlings displayed a significant biomass advantage over Grade II seedlings, and Grade II seedlings similarly outperformed Grade III seedlings. These results indicate that seedlings can continue to increase biomass over time and that biomass accumulation differs among growth stages. At the same time, compared with Grade II and Grade III seedlings, Grade I seedlings showed better growth adaptability and stronger biomass accumulation. Previous studies showed that aboveground biomass grew allometrically in early and middle stages but shifted to isometric growth in the late stage [30]. Later growth rates for both aboveground and belowground biomass were higher than earlier, indicating a change in growth allocation [30]. Grade I seedlings had significantly greater biomass in roots, stems, leaves, aboveground parts, and individual plants than Grades II and III [39]. Therefore, biomass varied among seedling grades.

### 3.2. The Allometric Growth Relationship Among the Biomass of Each Organ of P. yunnanensis Seedlings of Different Grades at Different Developmental Stages

As shown in Table 2 and Figure S2, at 240 days, *P. yunnanensis* seedlings showed weak allometric relationships among organs, with isometric growth as the dominant pattern. The biomass of root–stem, root–needle, and root–aboveground pairs showed significant correlations. These correlations differed significantly among grades. Grade I seedlings had the highest allometric curve position. This meant that, for each log value unit increase in root biomass, the log value increase in needle biomass was the greatest in Grade I. They had the highest efficiency of assimilate allocation to aboveground parts. They also had the strongest growth drive. Grade II seedlings had intermediate allometric exponents and intercepts between Grade I and Grade III. Their growth rate was moderate. Grade III seedlings had the lowest curve position. Their root expansion showed only a weak association with needle biomass accumulation. Their aboveground growth was clearly limited.

As shown in Table 3 and Figure S3, as development progressed to 330 days, allometric characteristics began to emerge among organs. The root–stem and root–needle biomass transitioned from isometric to allometric growth, with divergent growth rates and significant differences among size classes. The root–aboveground biomass showed distinct allometry, and growth synchrony between belowground and aboveground parts diminished. Stem–needle and stem–aboveground biomass showed reduced coordination and diverging allocation ratios. Although needle–aboveground biomass remained largely isometric, some size classes showed allometric tendencies, indicating that seedlings entered a stage of adjusting biomass allocation strategies. Among the grades, Grade I seedlings showed the clearest allometric pattern. For each log-unit increase in root investment, the stem biomass accumulation rate was the fastest, and its efficiency was the highest. This may suggest that Grade I seedlings had a stronger ability to synthesize and transport assimilates.

As shown in Table 4 and Figure S4, by 420 days, allometric relationships strengthened further. Root–stem and root–needle biomass exhibited obvious allometric characteristics with declining growth synchrony. Root–aboveground biomass showed significant allometric growth, and the belowground growth rate was generally lower than that of aboveground parts. Differences between stem–needle and stem–aboveground biomass enlarged, accompanied by reduced synchrony. The allometric pattern of needle–aboveground biomass gradually stabilized, and biomass allocation ratios converged, forming an allocation pattern characterized by priority accumulation in aboveground parts. This also indicated that Grade I seedlings reduced needle investment and shifted photosynthates to roots and stems, which is consistent with better resource uptake and support. Grade II was intermediate in the transition. Grade III had the most needles but the least root and stem biomass, still relying on leaf area but with low efficiency at 420 days.

As shown in Table 5 and Figure S5, subsequently, at 510 days, allometric relationships among organs stabilized and became statistically significant. Root–stem, root–needle, and root–aboveground biomass maintained stable allometric growth with obvious disparities between belowground and aboveground growth rates. Allocation patterns of stem–needle and stem–aboveground biomass became fixed, and the growth advantage of stems gradually became prominent. Needle–aboveground biomass maintained highly coordinated allometric relationships, with significant differences in slopes among pairs, indicating that the biomass allocation strategy was basically established. At this stage, Grade I seedlings showed the fastest increase in stem biomass and needle biomass. Their coordination and efficiency of assimilate allocation to stems were much better than those for other grades. Grade II had the next fastest allometric exponent. The stem growth was still positive but slowed greatly. Grade III stem biomass showed little change with needle biomass. This indicated that low-grade seedlings had clear disadvantages in organ growth coordination.

As shown in Table 6 and Figure S6, at 600 days, allometric relationships among organs reached highly significant levels. Allometric patterns of root, stem, needle, and aboveground biomass fractions were stable, and growth rate differences between belowground and aboveground parts were fully fixed. Allocation ratios of stem–needle and stem–aboveground biomass no longer changed with time. Needle–aboveground biomass showed the strongest coordination and highly significant allometry, indicating that plants had entered a mature, stable allocation stage. At the same log value of root or stem biomass, Grade I had the highest needle and root biomass, Grade II was next, and Grade III was the lowest. Higher grades prioritized photosynthates to needles and roots for better efficiency. Grade III had the least total biomass and low per-unit investment returns, showing poor reserves and weak uptake.

As shown in Table 7 and Figure S7, eventually, at 690 days, the allometric growth pattern was fully mature. For each of root, stem, needle, and aboveground biomass fractions, highly significant allometric relationships were detected, with growth rate differences held constant. Stem–needle and stem–aboveground biomass formed stable proportions unique to mature individuals, and needle contributed the most to aboveground biomass. Needle–aboveground biomass maintained highly coordinated allometric growth; the next most coordinated growth was for root biomass. The overall allocation strategy was consistent with that of mature plants, and the allometric growth pattern was finally finalized. Among them, Grade I seedlings supported larger stem and needle biomass. Their root efficiency was the highest. This indicates that Grade I seedlings exhibited distinct allometric relationships between organs and individual biomass.

In contrast, Grade III seedlings were at a disadvantage. Their organ growth rates were slower. In summary, Grade I seedlings built a more efficient assimilate allocation network at 690 days. Their overall growth was significantly better than Grade II and Grade III. Previous studies have investigated the seasonal morphological variations of *P. yunnanensis* seedlings of different grades and also found that the biomass of Grade I, Grade II, and Grade III seedlings exhibited a progressive increase, reaching a peak at the later growth stage [40]. Needles accounted for the highest biomass allocation, followed by roots, and the higher the grade, the higher the accumulation rate of biomass allocation [40].

Overall, as seedlings grew, allometric patterns and organ allocation strategies changed. They showed a dynamic process from initial establishment to optimization and then stability. The different grades showed clear differences. Grade I seedlings always kept the highest biomass accumulation rate and the most flexible allocation plasticity. Results showed that Grade I seedlings had the highest allometric trajectory position from day 240 onward, with the greatest needle gain per-unit root investment, reflecting inherent morphological advantages. After day 330, they showed the fastest accumulation rates in stems and needles. By day 420, they actively adjusted allocation strategies, reducing needle allocation while enhancing root and stem allocation. From day 510 to 690, they consistently maintained the most coordinated organ growth and the highest allocation efficiency. At the same time, this showed they have innate resource advantages. They can adjust allometric relationships to optimize carbon economy under environmental changes. Thus, they had stronger early competitiveness and survival potential in afforestation.

### 3.3. Allometric Growth of Organ and Individual Plant Biomass in P. yunnanensis Seedlings Across Grades and Stages

As shown in Table 8 and Figure S8, at 240 days, root–individual, stem–individual, needle–individual, and aboveground–individual biomass showed significant correlations in *P. yunnanensis* seedlings, with significant differences among size classes. Isometric growth was dominant. Organ biomass increased synchronously and evenly with individual biomass, and contributions from belowground and aboveground parts were similar, indicating coordinated and stable growth without obvious allocation bias. At the same biomass level, growth and allocation strategies differ among grades. Grade I seedlings allocate more photosynthates to roots, needles, and aboveground parts. Grade II shows lower allocation efficiency, and Grade III has the lowest absolute increment in all organs. Thus, by 240 days, Grade I seedlings showed a distinct allometric pattern, which may be associated with stronger competitiveness and environmental adaptability. Therefore, Grade I seedlings established the optimal allometric pattern. This showed stronger interspecific competitiveness and environmental adaptability at 240 days.

As shown in Table 9 and Figure S9, as development progressed to 330 days, allometric growth between organs and individual biomass began to appear. Root–individual biomass shifted from isometric to allometric growth, with reduced synchrony and slower belowground accumulation relative to the whole plant. Stem–individual, needle–individual, and aboveground–individual remained largely isometric, but slopes diverged among size classes, indicating the onset of differential resource allocation. Grade I seedlings had the largest root, stem, needle, and aboveground biomass accumulation. Grade III seedlings always had the lowest levels. Their root and leaf construction was limited by overall growth potential.

As shown in Table 10 and Figure S10, by 420 days, allometric relationships strengthened further. Root–individual biomass showed obvious allometric growth, with the belowground growth rate persistently lower than that of the whole plant. Stem–individual and aboveground–individual displayed emerging allometric patterns, with stem growth faster than the average. Needle–individual synchrony decreased, and differences among size classes intensified, leading to a clear divergence in organ contributions. Moreover, the Grade I seedlings showed more sensitive responses in root, stem, needle, and aboveground biomass accumulation to per-plant biomass changes. With the same individual plant increment, Grade I increased organ biomass faster. Grade II and III might show unbalanced trends, which could be consistent with resource competition or stress. These trends included lower root proportion, redundant stem–needle growth, or excessive elongation of aboveground parts.

As shown in Table 11 and Figure S11, subsequently, at 510 days, all organs exhibited stable, significant allometric growth relative to individual biomass. Root accumulation was notably slower than the whole-plant rate. Stem–individual had a higher slope, indicating an increasing contribution to total biomass. Needle–individual and aboveground–individual maintained stable allometry with strong synchrony. Allocation proportions gradually stabilized, supporting subsequent growth. At the same time, Grade I seedlings showed the best quality traits in the growth trend. Their slope for aboveground biomass was the largest at 1.079. This means that as individual plant biomass increased, Grade I seedlings allocated more biomass to the stem within the aboveground part. The slope for stem allocation reached 1.097. This promoted rapid expansion of the crown and photosynthetic area. In contrast, Grade III seedlings showed the lowest organ biomass at the same individual plant size at 510 days.

As shown in Table 12 and Figure S12, at 600 days, allometric relationships became highly significant, and allocation patterns matured. Root–individual allometry was stable, with fixed growth disparities. Slopes of stem–individual and needle–individual differed significantly, and stem proportion increased further. Aboveground–individual maintained strong allometric synchrony, and allocation strategy changed little over time. Moreover, the Grade I seedlings occupied the highest positions in the models for root, stem, needle, and aboveground biomass. This meant that, at the same individual plant biomass level, they had higher baseline organ biomass accumulation. Their growth foundation was the best, and their allocation pattern was the most mature. Grade II seedlings showed relatively steady transitional growth. Grade III seedlings lagged significantly behind Grade I and Grade II. Therefore, Grade I seedlings showed a clear advantage in growth size, resource reserves, and overall development quality at 600 days.

As shown in Table 13 and Figure S13, eventually, at 690 days, allometric growth patterns between organs and individual biomass were fully mature. All pairs showed highly significant allometry with stable differences among size classes. Belowground growth was significantly slower than individual plant growth, while aboveground parts dominated biomass accumulation. Needle contribution was most prominent, forming a stable allocation strategy consistent with mature plants. At the same time, Grade I had the highest absolute organ biomass, deep roots, strong aboveground parts, and stable growth. Grade II was balanced. Grade III had higher slopes for needle and aboveground biomass but started too low. Their absolute biomass still fell behind Grade I and Grade II at 690 days.

Previous studies found that significant differences were observed among the biomass of various organs and individual plants [9]. The aboveground biomass exhibited an allometric relationship with individual plant biomass in Grade I seedlings, whereas Grade II and Grade III seedlings showed an isometric relationship [9]. This indicated that Grade I seedlings had a higher growth rate compared with Grade II and Grade III seedlings. In summary, as seedlings continued to grow, the allometric relationships and resource allocation strategies between different organs and total biomass changed over time. They also showed a dynamic process from initial establishment to gradual maturity. Notably, significant differences appeared among quality grades. Grade I seedlings had higher growth rates and stronger resource acquisition ability. Their allometric exponents and allocation slopes were much higher than those of lower grades. This showed their advantages in biomass allocation and allometric coordination. These findings reveal the key role of seedling quality in planting success. They also provide physiological and ecological evidence for selecting quality seedlings and managing target trees in plantation cultivation.

## 4. Discussion

### 4.1. Effects of Seedling Grade at Different Developmental Stages on Biomass Accumulation

Biomass accumulation is a core indicator for evaluating seedling growth potential and adaptability [41,42]. It is widely used in forest seedling cultivation research. Numerous studies have confirmed that there are differences in biomass among different grade levels [43,44]. In this study, all seedlings were cultivated under the same conditions. The biomass of individual plants in *P. yunnanensis* seedlings increased extremely significantly at different developmental stages from 240 days to 690 days under natural conditions, and Grade I seedlings maintained significantly higher organ biomass than Grade II and III seedlings. The biomass differences among seedling grades widened continuously as seedling age increased. Consistent with this, previous studies have also found that, as seedlings grow and develop, biomass accumulates over time [45]. Moreover, the Grade I seedlings had greater growth, higher yield, and better quality. They are the best seedlings for cultivation [45,46]. This indicates that early size hierarchies were associated with persistent biomass differences among grades.

Moreover, Grade I seedlings may have a larger initial photosynthetic area of needles, which could contribute to greater carbon supply for dry matter accumulation. Research indicated that during the fast-growing phase, Grade I seedlings exhibited the highest chlorophyll content [47]. At the molecular level, a previous study reported that genes associated with chlorophyll synthesis were markedly upregulated in Grade I seedlings, while degradation-related genes were more active in Grade III seedlings [47]. These findings suggest that weak seedlings may have limited photosynthetic capacity in foliage and that insufficient carbon assimilation may further restrict biomass production. This suggested that the growth advantage derived from individual size may be a common characteristic of plant seedlings. At the same time, Grade I seedlings had significantly higher biomass than Grades II and III at all stages, with the gap widening over time, confirming that the initial size hierarchy was amplified during the ontogeny of *P. yunnanensis* [19]. Long-term studies on *P. taeda* and *P. elliottii* similarly showed that higher-grade seedlings maintained advantages in survival and growth [27]. In this study, Grade I also exhibited more stable allometric relationships and better organ coordination, reflecting inherent advantages in resource use and allocation that were progressively amplified. Therefore, the initial seedling hierarchy is not erased but persists over development, highlighting the long-term value of selecting superior seedlings in afforestation. Therefore, Grade I seedlings are regarded as superior in terms of total biomass.

### 4.2. Dynamic Changes in Organ Biomass Accumulation and Growth Differences Among Seedling Grades

Biomass accumulation in various seedling organs differed greatly across growth stages. Needles dominated the biomass composition and presented a steady increasing trend. The root biomass increased rapidly during the mid-to-late growth phases, and the stem biomass grew slowly and made a prominent contribution to biomass allocation. The previous studies showed that plants increased allocation to stems [48]. This increase was limited, but it helped raise the canopy for light competition [48]. The previous study also investigated the biomass characteristics and allometric relationships of three-year-old *Juglans mandshurica* seedlings originating from 21 seed sources across China [49]. It found that biomass allocation patterns vary among different geographic sources [49]. Other researchers studied biomass allocation in seedlings of six broadleaf tree species [50]. They found that more biomass was given to the aboveground parts [50]. Collectively, these results further support the optimal allocation theory. Plant individuals preferentially allocate resources to photosynthetic organs to sustain carbon assimilation and facilitate biomass accumulation at the individual level.

As individuals grow, water and nutrient acquisition become limiting, and investment in roots and stems increases accordingly. Researchers found that biomass allocation is co-regulated by species, ontogenetic stage, and environment [15]. Although plants have limited ability to adjust allocation, changes in allocation during ontogeny are a common ecological phenomenon [15]. Meanwhile, the disparity in accumulation rates among grades increased over time. Grade I seedlings sustained superior growth performance, whereas Grade II and Grade III seedlings showed inferior growth status. This indicates that initial phenotypic differences will be magnified during subsequent development. Previous studies have shown that total biomass increases with seedling grade [20,51]. Allometric and isometric relationships among organs changed with grade [31]. Higher-grade seedlings had stronger coordinative plasticity [31]. This was associated with accelerated allometric growth, and seedling growth exhibited a distinct grade-dependent decline from Grade I to Grade III [9,31]. At the same biomass level, Grade I seedlings may have higher chlorophyll content and may allocate more photosynthates to roots, needles, and aboveground parts, which is consistent with greater biomass accumulation efficiency and stronger carbon assimilation capacity [29,42]. Among them, Grade I outperformed the other grades in growth performance. Our results agree with previous studies. We further showed that biomass allocation among organs differed across growth stages. Aboveground biomass contributes prominently to the whole plant, with needle biomass contributing the most. The developmental stage also differed significantly among grades. Grade I showed the most obvious advantages across stages. Therefore, needles dominate the biomass composition during seedling growth. Higher-grade seedlings had the most significant growth and biomass allocation.

### 4.3. Allometric Growth Dynamics of Seedling Organs

Analysis of allometric growth correlations is a vital approach for exploring the relative growth characteristics of plant organs and clarifying adaptive strategies in natural habitats [52]. Because organ biomass is a component of individual biomass, these organ-to-total regressions involve mathematical coupling. Therefore, the relationships reported here are interpreted as descriptive allometric patterns rather than as direct evidence of physiological coordination. Specifically, the temporally variable allometric patterns observed in *P. yunnanensis* seedlings may represent adaptive responses to changing resource availability during growth. Notably, in early development, seedlings mainly showed isometric growth. All organs grew in coordination. Significant allometric growth appeared gradually in the middle and late stages. Organ growth rates also diverged clearly at that time. At 510 days, seedling organ growth and biomass allocation started to mature. At 600 days, they reached a preliminary mature state. At 690 days, they became fully mature. The previous studies on *P. yunnanensis* and other vegetation found that, as seedlings developed, the growth type shifted from isometric to allometric, and the growth state gradually matured [21,29,46]. This temporal pattern matches our results. Furthermore, this shift from isometric to allometric growth was essentially a result of differential relative growth rates among organs during ontogeny. In the early stage, organs grew coordinately, helping to quickly establish a functional framework. As size increased, physiological functions and resource efficiency diverged, growth rates differentiated, and a stable allometric pattern eventually emerged.

Seedling development showed how individual growth changed across stages. Researchers studying alpine plants found that, after controlling for individual size, seedling growth still had a significant independent effect on biomass allocation, indicating that developmental stage effects cannot be fully attributed to changes in plant size [2]. Seedlings first built functional organs in a balanced way. Then they allocated more photosynthates to dominant organs. This helped improve resource acquisition. This study integrated multi-organ coordination information from roots, stems, and leaves across spatial scales.

In summary, the ontogenetic changes in biomass allocation patterns of *P. yunnanensis* seedlings, the shift from isometric to allometric growth, and the continuous amplification of initial grade differences are intrinsically linked. Previous studies have revealed that linking traits, ontogenetic stages, and population dynamics is key to understanding community assembly mechanisms. These findings strengthen the experimental evidence base for growth and development patterns in subtropical pine seedlings with greater continuity and comprehensiveness. The results indicated that seedling grade is not merely a static quality classification but rather an important biological variable that is associated with ontogenetic trajectories and long-term growth performance [53]. The study also revealed the stage-specific changes in allometric exponents and their interactions with seedling grades. This provides strong data support for the adaptive trade-off hypothesis in early regeneration strategies for subtropical evergreen conifers. Forest managers can then make precise tending decisions based on growth stage and seedling grade.

## 5. Limitations and Future Research Prospects

This study still has some limitations. First, this study monitored seedlings only under natural growth conditions. It did not consider the regulatory effects of environmental factors such as light, water, and nutrients, so the research conditions were relatively simple. Second, the experimental period ranged from 240 to 690 days, mainly covering the seedling stage, and did not include long-term growth performance after afforestation. We recognize that organ-to-total biomass regressions involve mathematical coupling, so significant correlations may reflect structure more than physiology. As we log-transformed the data and focused on relative growth relationships, our results are used solely to describe relative growth rates. In addition, this study analyzed only biomass and allometric growth, without further exploration using physiological and biochemical indices and molecular mechanisms. 

In future experiments, we can set different time intervals. We can quantify the allometric growth dynamics of graded seedlings. We can also identify key time periods for shifts in organ allocation. Long-term plantation plot monitoring clarifies connections between seedling allometric traits and young-stand productivity. Physiological tests combined with transcriptome analysis reveal growth differentiation mechanisms of different seedling grades. We can also cooperate with breeding teams to screen superior *P. yunnanensis* families.

## 6. Conclusions

Under natural conditions, we graded annual *P. yunnanensis* seedlings into three quality classes. We then systematically charted the temporal dynamics of organ biomass gain, partitioning, and allometric scaling over six successive growth stages from 240 to 690 days. As seedlings grow and develop, organ biomass increases significantly, and allometric growth patterns shift accordingly. Needles dominate biomass accumulation throughout the entire growth period. In the early phase of development, isometric growth prevailed among organs. However, this pattern shifted to allometric growth in the mid-to-late stages, accompanied by progressively widening growth rate disparities. The allometric patterns became fully mature, forming a stable allocation strategy from 510 days to 690 days. Most importantly, biomass accumulation and allometric growth differed among seedling grades across developmental stages. Meanwhile, Grade I seedlings exhibited significant advantages in biomass accumulation, with more stable allometric relationships and stronger organ coordination, whereas Grade II and Grade III seedlings showed more-pronounced fluctuations. In summary, seedlings of different grades differed in biomass accumulation dynamics and allometric growth patterns across various developmental stages, which may represent important adaptive strategies for *P. yunnanensis* seedlings in natural environments.

## Figures and Tables

**Figure 1:**
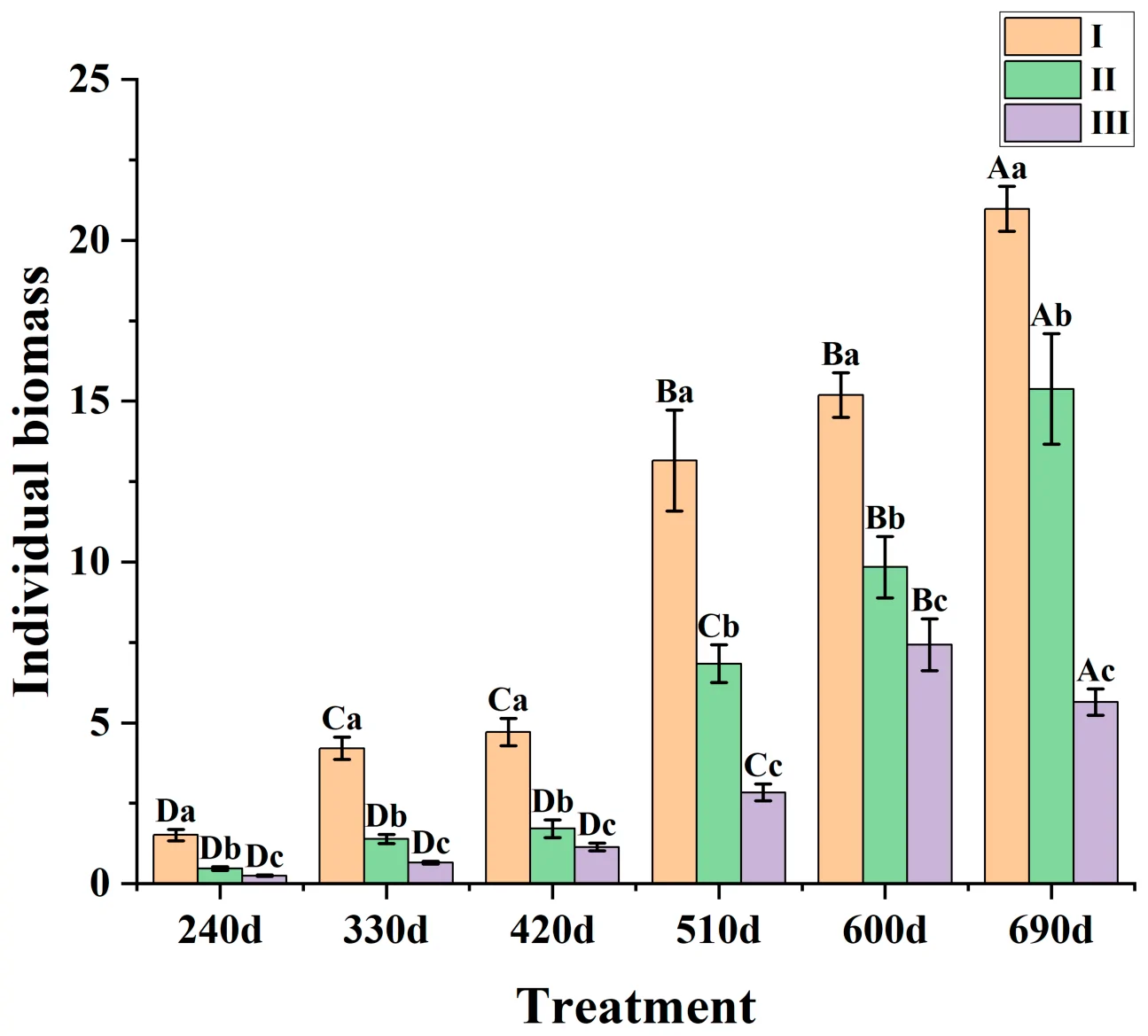
The variation in individual biomass of seedlings with different grades at different developmental stages from 240 days to 690 days. Capital letters indicate significant differences among different developmental stages within the same seedling grade, while the distinct lowercase letters represent significant differences between seedling grades at a different developmental stage.

**Table 1:** The two-way ANOVA of the effects of seedling grade and developmental stage on individual biomass.

Factors	Degree of Freedom	Relevant Test Statistics	*p*	Partial Eta Squared
Grade	2	146.768	*p* < 0.001	0.671
Stage	5	173.264	*p* < 0.001	0.857
Grade × Stage	10	14.935	*p* < 0.001	0.509

**Table 2:** Allometric analysis of organ biomass among different grades of annual *P. yunnanensis* seedlings at 240 days (Note: Relationships classified as allometric (A) or isometric (I)).

Stages	x	y	Grade	*n*	R^2^	Slope	Interc	F	*p*	Type
240 days	Log (Root biomass)	Log (Stem biomass)	I	9	0.696	1.04981	−0.13753	0.001	0.975	I
			II	9	0.722	1.26045	0.04954	0.859	0.385	I
			III	9	0.394	0.88322	−0.3231	0.021	0.888	I
240 days	Log (Root biomass)	Log (Needle biomass)	I	9	0.671	0.73798	0.31229	2.541	0.155	I
			II	9	0.892	1.24428	0.69041	1.515	0.258	I
			III	9	0.718	0.52143	−0.20751	14.181	0.007	A
240 days	Log (Root biomass)	Log (Aboveground biomass)	I	9	0.719	0.79739	0.43931	1.909	0.210	I
			II	9	0.884	1.23128	0.76456	1.209	0.308	I
			III	9	0.667	0.58519	0.02162	7.605	0.028	A
240 days	Log (Stem biomass)	Log (Needle biomass)	I	9	0.746	0.74575	0.43954	3.433	0.106	I
			II	9	0.816	1.03239	0.69875	0.038	0.852	I
			III	9	0.608	0.52788	−0.10301	8.836	0.021	A
240 days	Log (Stem biomass)	Log (Aboveground biomass)	I	9	0.862	0.80750	0.57751	4.102	0.082	I
			II	9	0.876	1.02479	0.77636	0.101	0.760	I
			III	9	0.815	0.62883	0.14640	11.545	0.011	A
240 days	Log (Needle biomass)	Log (Aboveground biomass)	I	9	0.978	1.11597	0.10858	1.377	0.279	I
			II	9	0.993	1.01714	0.09729	0.116	0.744	I
			III	9	0.947	1.20512	0.28161	2.158	0.185	I

**Table 3:** Allometric analysis of organ biomass among different grades of annual *P. yunnanensis* seedlings at 330 days (Note: Relationships classified as allometric (A) or isometric (I)).

Stages	x	y	Grade	*n*	R^2^	Slope	Interc	F	*p*	Type
330 days	Log (Root biomass)	Log (Stem biomass)	I	9	0.787	0.68318	−0.08421	6.790	0.035	A
			II	9	0.312	0.51053	−0.31278	3.208	0.116	I
			III	9	0.276	0.83427	−0.26943	0.002	0.984	I
330 days	Log (Root biomass)	Log (Needle biomass)	I	9	0.020	0.2756	0.37739	0.222	0.652	I
			II	9	0.839	0.7624	0.18740	5.118	0.058	I
			III	9	0.560	0.93657	0.23346	0.066	0.804	I
330 days	Log (Root biomass)	Log (Aboveground biomass)	I	9	0.057	0.40927	0.49698	1.196	0.310	I
			II	9	0.837	0.66312	0.29413	10.482	0.014	A
			III	9	0.668	0.79424	0.26781	1.514	0.258	I
330 days	Log (Stem biomass)	Log (Needle biomass)	I	9	0.042	0.30713	0.35207	3.139	0.120	I
			II	9	0.299	1.05340	0.42209	0.400	0.547	I
			III	9	0.080	0.61409	0.08159	0.018	0.896	I
330 days	Log (Stem biomass)	Log (Aboveground biomass)	I	9	0.029	0.57150	0.52636	0.018	0.898	I
			II	9	0.517	1.04177	0.56735	0.051	0.827	I
			III	9	0.365	0.70537	0.30502	0.701	0.430	I
330 days	Log (Needle biomass)	Log (Aboveground biomass)	I	9	0.846	0.60614	0.33998	16.173	0.005	A
			II	9	0.950	0.92138	0.14393	2.742	0.142	I
			III	9	0.874	0.87495	0.08407	2.264	0.176	I

**Table 4:** Allometric analysis of organ biomass among different grades of annual *P. yunnanensis* seedlings at 420 days (Note: Relationships classified as allometric (A) or isometric (I)).

Stages	x	y	Grade	*n*	R^2^	Slope	Interc	F	*p*	Type
420 days	Log (Root biomass)	Log (Stem biomass)	I	9	0.014	0.42446	0.19051	0.159	0.702	I
			II	9	0.690	0.98611	−0.18465	0.067	0.804	I
			III	9	0.274	1.08054	−0.15237	0.593	0.466	I
420 days	Log (Root biomass)	Log (Needle biomass)	I	9	0.235	0.79956	0.18231	0.003	0.955	I
			II	9	0.740	0.80563	−0.05652	1.981	0.202	I
			III	9	0.736	1.64728	0.19029	5.693	0.048	A
420 days	Log (Root biomass)	Log (Aboveground biomass)	I	9	0.163	0.59893	0.47581	0.431	0.533	I
			II	9	0.769	0.84751	0.17919	1.525	0.257	I
			III	9	0.760	1.29613	0.30612	1.230	0.304	I
420 days	Log (Stem biomass)	Log (Needle biomass)	I	9	0.313	0.99366	0.17615	0.173	0.690	I
			II	9	0.696	0.84217	0.11364	1.066	0.336	I
			III	9	0.182	0.94106	0.10891	0.356	0.569	I
420 days	Log (Stem biomass)	Log (Aboveground biomass)	I	9	0.684	0.96368	0.40594	0.127	0.732	I
			II	9	0.898	0.90303	0.36284	1.949	0.205	I
			III	9	0.596	0.97089	0.36943	0.030	0.867	I
420 days	Log (Needle biomass)	Log (Aboveground biomass)	I	9	0.859	0.86994	0.35259	2.131	0.188	I
			II	9	0.934	1.11296	0.25930	0.219	0.654	I
			III	9	0.810	0.83392	0.18102	2.237	0.178	I

**Table 5:** Allometric analysis of organ biomass among different grades of annual *P. yunnanensis* seedlings at 510 days (Note: Relationships classified as allometric (A) or isometric (I)).

Stages	x	y	Grade	*n*	R^2^	Slope	Interc	F	*p*	Type
510 days	Log (Root biomass)	Log (Stem biomass)	I	9	0.593	0.77709	0.23012	1.220	0.306	I
			II	9	0.244	1.00356	0.00559	0.371	0.562	I
			III	9	0.287	0.77810	−0.18770	0.087	0.777	I
510 days	Log (Root biomass)	Log (Needle biomass)	I	9	0.656	0.82968	0.66153	0.983	0.354	I
			II	9	0.771	0.91215	0.47347	0.687	0.434	I
			III	9	0.685	0.92809	0.41860	0.289	0.608	I
510 days	Log (Root biomass)	Log (Aboveground biomass)	I	9	0.687	0.79921	0.79118	1.591	0.248	I
			II	9	0.634	0.92440	0.60089	0.204	0.665	I
			III	9	0.715	0.86631	0.50935	0.895	0.376	I
510 days	Log (Stem biomass)	Log (Needle biomass)	I	9	0.705	1.09337	0.59895	0.049	0.831	I
			II	9	0.681	0.73326	0.59497	2.767	0.140	I
			III	9	0.197	0.76754	0.50233	0.003	0.955	I
510 days	Log (Stem biomass)	Log (Aboveground biomass)	I	9	0.833	1.06680	0.69058	0.000	0.998	I
			II	9	0.826	0.78852	0.69419	3.778	0.093	I
			III	9	0.374	0.82735	0.62683	0.103	0.758	I
510 days	Log (Needle biomass)	Log (Aboveground biomass)	I	9	0.976	0.99946	0.22692	0.620	0.457	I
			II	9	0.971	1.09922	0.12873	0.519	0.494	I
			III	9	0.960	0.97793	0.14198	1.023	0.345	I

**Table 6:** Allometric analysis of organ biomass among different grades of annual *P. yunnanensis* seedlings at 600 days (Note: Relationships classified as allometric (A) or isometric (I)).

Stages	x	y	Grade	*n*	R^2^	Slope	Interc	F	*p*	Type
600 days	Log (Root biomass)	Log (Stem biomass)	I	9	0.441	0.83384	0.33772	0.203	0.666	I
			II	9	0.010	0.31624	0.37307	1.224	0.305	I
			III	9	0.887	0.85808	−0.03119	3.027	0.125	I
600 days	Log (Root biomass)	Log (Needle biomass)	I	9	0.010	0.32223	1.01053	1.119	0.325	I
			II	9	0.461	1.22785	0.52661	0.827	0.393	I
			III	9	0.736	0.58803	0.51441	10.026	0.016	A
600 days	Log (Root biomass)	Log (Aboveground biomass)	I	9	0.144	0.40708	1.04957	3.112	0.121	I
			II	9	0.588	0.84670	0.70694	0.531	0.490	I
			III	9	0.809	0.64143	0.62314	10.250	0.015	A
600 days	Log (Stem biomass)	Log (Needle biomass)	I	9	0.010	0.36665	1.01695	0.493	0.505	I
			II	9	0.075	0.17098	1.07951	3.735	0.095	I
			III	9	0.865	0.74423	0.54131	7.105	0.032	A
600 days	Log (Stem biomass)	Log (Aboveground biomass)	I	9	0.270	0.53483	0.99279	2.144	0.187	I
			II	9	0.000	0.48171	1.00533	0.374	0.560	I
			III	9	0.938	0.79754	0.65285	9.513	0.018	A
600 days	Log (Needle biomass)	Log (Aboveground biomass)	I	9	0.811	0.87598	0.51049	1.426	0.271	I
			II	9	0.914	0.69718	0.47228	15.688	0.005	A
			III	9	0.985	1.12024	0.11630	2.553	0.154	I

**Table 7:** Allometric analysis of organ biomass among different grades of annual *P. yunnanensis* seedlings at 690 days (Note: Relationships classified as allometric (A) or isometric (I)).

Stages	x	y	Grade	*n*	R^2^	Slope	Interc	F	*p*	Type
690 days	Log (Root biomass)	Log (Stem biomass)	I	9	0.091	0.69321	0.77505	0.016	0.901	I
			II	9	0.224	0.37738	0.49219	6.288	0.041	A
			III	9	0.046	0.37583	0.15126	1.513	0.258	I
690 days	Log (Root biomass)	Log (Needle biomass)	I	9	0.120	0.36405	0.95745	3.922	0.088	I
			II	9	0.619	1.20821	0.31495	0.532	0.490	I
			III	9	0.442	0.48875	0.31421	6.330	0.040	A
690 days	Log (Root biomass)	Log (Aboveground biomass)	I	9	0.358	0.37042	1.05943	11.125	0.012	A
			II	9	0.620	0.83265	0.66425	0.774	0.408	I
			III	9	0.246	0.44492	0.53992	4.008	0.085	I
690 days	Log (Stem biomass)	Log (Needle biomass)	I	9	0.119	0.00475	1.20518	4.585	0.070	I
			II	9	0.195	1.97669	0.63311	9.926	0.016	A
			III	9	0.698	0.83591	0.30988	1.158	0.318	I
690 days	Log (Stem biomass)	Log (Aboveground biomass)	I	9	0.439	0.37120	1.05011	14.474	0.007	A
			II	9	0.361	1.57499	0.70058	3.754	0.094	I
			III	9	0.887	0.89537	0.48294	1.956	0.205	I
690 days	Log (Needle biomass)	Log (Aboveground biomass)	I	9	0.222	0.63649	1.05372	0.503	0.501	I
			II	9	0.960	0.72709	0.50051	25.200	0.002	A
			III	9	0.944	1.10916	0.22454	0.249	0.633	I

**Table 8:** Allometric analysis between organ biomass and individual biomass among different grades of annual *P. yunnanensis* seedlings at 240 days (Note: Relationships classified as allometric (A) or isometric (I)).

Stages	x	y	Grade	*n*	R^2^	Slope	Interc	F	*p*	Type
240 days	Log (Root biomass)	Log (Individual biomass)	I	9	0.851	0.84522	0.57461	2.633	0.149	I
			II	9	0.925	1.17917	0.82459	0.962	0.359	I
			III	9	0.849	0.68290	0.22272	9.910	0.016	A
240 days	Log (Stem biomass)	Log (Individual biomass)	I	9	0.862	0.83977	0.71169	3.007	0.127	I
			II	9	0.859	0.98450	0.83995	0.336	0.580	I
			III	9	0.703	0.69691	0.36757	4.077	0.083	I
240 days	Log (Needle biomass)	Log (Individual biomass)	I	9	0.942	1.17887	0.22785	1.344	0.284	I
			II	9	0.992	0.97968	0.18896	2.119	0.189	I
			III	9	0.943	1.36372	0.54100	7.833	0.027	A
240 days	Log (Aboveground biomass)	Log (Individual biomass)	I	9	0.974	1.09005	0.12017	0.407	0.544	I
			II	9	0.995	0.98413	0.10561	2.243	0.178	I
			III	9	0.954	1.19586	0.27026	2.286	0.174	I

**Table 9:** Allometric analysis between organ biomass and individual biomass among different grades of annual *P. yunnanensis* seedlings at 330 days (Note: Relationships classified as allometric (A) or isometric (I)).

Stages	x	y	Grade	*n*	R^2^	Slope	Interc	F	*p*	Type
330 days	Log (Root biomass)	Log (Individual biomass)	I	9	0.555	0.59601	0.61570	4.547	0.070	I
			II	9	0.933	0.75012	0.46862	13.262	0.008	A
			III	9	0.843	0.85136	0.45096	2.334	0.170	I
330 days	Log (Stem biomass)	Log (Individual biomass)	I	9	0.396	0.85739	0.69136	0.064	0.808	I
			II	9	0.444	1.14199	0.75942	0.441	0.528	I
			III	9	0.371	0.73578	0.47297	0.505	0.500	I
330 days	Log (Needle biomass)	Log (Individual biomass)	I	9	0.291	0.43014	0.59017	5.489	0.052	I
			II	9	0.949	1.04630	0.30117	0.022	0.887	I
			III	9	0.821	0.90645	0.23982	1.039	0.342	I
330 days	Log (Aboveground biomass)	Log (Individual biomass)	I	9	0.675	0.91680	0.36752	0.329	0.584	I
			II	9	0.976	1.18805	0.14611	4.673	0.067	I
			III	9	0.958	1.09754	0.17372	0.241	0.638	I

**Table 10:** Allometric analysis between organ biomass and individual biomass among different grades of annual *P. yunnanensis* seedlings at 420 days (Note: Relationships classified as allometric (A) or isometric (I)).

Stages	x	y	Grade	*n*	R^2^	Slope	Interc	F	*p*	Type
420 days	Log (Root biomass)	Log (Individual biomass)	I	9	0.580	0.74664	0.59676	1.533	0.256	I
			II	9	0.919	0.90021	0.40120	2.499	0.158	I
			III	9	0.885	1.18186	0.47890	0.615	0.459	I
420 days	Log (Stem biomass)	Log (Individual biomass)	I	9	0.404	0.79850	0.63405	0.264	0.623	I
			II	9	0.861	0.95274	0.59960	0.668	0.441	I
			III	9	0.506	0.84484	0.51541	0.301	0.600	I
420 days	Log (Needle biomass)	Log (Individual biomass)	I	9	0.785	0.80244	0.56219	2.615	0.150	I
			II	9	0.900	1.17888	0.49189	0.682	0.436	I
			III	9	0.832	0.75347	0.36267	5.241	0.056	I
420 days	Log (Aboveground biomass)	Log (Individual biomass)	I	9	0.808	0.98883	0.34111	0.199	0.669	I
			II	9	0.955	1.11511	0.22973	0.441	0.528	I
			III	9	0.973	0.94553	0.20943	2.751	0.141	I

**Table 11:** Allometric analysis between organ biomass and individual biomass among different grades of annual *P. yunnanensis* seedlings at 510 days (Note: Relationships classified as allometric (A) or isometric (I)).

Stages	x	y	Grade	*n*	R^2^	Slope	Interc	F	*p*	Type
510 days	Log (Root biomass)	Log (Individual biomass)	I	9	0.824	0.84325	0.84692	2.226	0.179	I
			II	9	0.781	0.94047	0.69749	0.465	0.517	I
			III	9	0.842	0.89375	0.62661	1.417	0.273	I
510 days	Log (Stem biomass)	Log (Individual biomass)	I	9	0.819	1.09676	0.79537	0.032	0.864	I
			II	9	0.697	0.75805	0.82356	2.438	0.162	I
			III	9	0.371	0.83557	0.74298	0.079	0.787	I
510 days	Log (Needle biomass)	Log (Individual biomass)	I	9	0.947	1.04270	0.34984	0.037	0.854	I
			II	9	0.995	1.05485	0.23046	1.128	0.323	I
			III	9	0.937	0.99661	0.25990	0.462	0.518	I
510 days	Log (Aboveground biomass)	Log (Individual biomass)	I	9	0.974	1.07847	0.15380	0.229	0.647	I
			II	9	0.973	1.03039	0.18609	0.078	0.789	I
			III	9	0.976	1.05873	0.13338	0.041	0.845	I

**Table 12:** Allometric analysis between organ biomass and individual biomass among different grades of annual *P. yunnanensis* seedlings at 600 days (Note: Relationships classified as allometric (A) or isometric (I)).

Stages	x	y	Grade	*n*	R^2^	Slope	Interc	F	*p*	Type
600 days	Log (Root biomass)	Log (Individual biomass)	I	9	0.495	0.55184	1.04213	4.935	0.062	I
			II	9	0.744	0.87461	0.78424	0.962	0.359	I
			III	9	0.921	0.71706	0.71770	14.738	0.006	A
600 days	Log (Stem biomass)	Log (Individual biomass)	I	9	0.462	0.61669	1.04385	2.674	0.146	I
			II	9	0.002	0.51832	1.11117	0.340	0.578	I
			III	9	0.963	0.88338	0.76902	5.935	0.045	A
600 days	Log (Needle biomass)	Log (Individual biomass)	I	9	0.531	0.82637	0.82318	0.479	0.511	I
			II	9	0.858	0.69104	0.62474	9.997	0.016	A
			III	9	0.930	1.28104	0.23341	3.491	0.104	I
600 days	Log (Aboveground biomass)	Log (Individual biomass)	I	9	0.854	0.93982	0.18436	0.014	0.909	I
			II	9	0.972	1.03795	0.19500	0.028	0.871	I
			III	9	0.972	1.17183	0.10906	3.003	0.127	I

**Table 13:** Allometric analysis between organ biomass and individual biomass among different grades of annual *P. yunnanensis* seedlings at 690 days (Note: Relationships classified as allometric (A) or isometric (I)).

Stages	x	y	Grade	*n*	R^2^	Slope	Interc	F	*p*	Type
690 days	Log (Root biomass)	Log (Individual biomass)	I	9	0.826	0.47866	0.93172	18.943	0.003	A
			II	9	0.866	0.89863	0.70072	1.588	0.248	I
			III	9	0.696	0.61748	0.64757	6.915	0.034	A
690 days	Log (Stem biomass)	Log (Individual biomass)	I	9	0.277	0.42619	1.24611	5.342	0.054	I
			II	9	0.342	1.60176	0.89919	4.091	0.083	I
			III	9	0.495	0.89379	0.70572	0.098	0.763	I
690 days	Log (Needle biomass)	Log (Individual biomass)	I	9	0.203	0.79830	1.21121	0.002	0.970	I
			II	9	0.890	0.75848	0.72046	7.797	0.027	A
			III	9	0.930	1.22124	0.38479	1.910	0.209	I
690 days	Log (Aboveground biomass)	Log (Individual biomass)	I	9	0.770	1.12399	0.01903	1.904	0.210	I
			II	9	0.918	1.09876	0.31004	0.076	0.791	I
			III	9	0.796	1.16657	0.30236	0.298	0.602	I

## Data Availability

All data generated or analyzed during this study are included in this article. All data generated or analyzed during this study are available from the first author or request.

## Supplementary Materials

The following supporting information can be downloaded at https://www.mdpi.com/article/10.3390/biology15191692/s1: Figure S1: Morphological comparison of *P. yunnanensis* seedlings across three quality grades, where (A) denotes Grade I, (B) denotes Grade II, and (C) denotes Grade III [31]; Figures S2–S7: Allometric relationships among organ biomasses of different *P. yunnanensis* grades at 240, 330, 420, 510, 600, and 690 days, respectively. Figures S8–S13: Allometric relationships of root, stem, needle, and aboveground biomass to individual plant biomass at 240, 330, 420, 510, 600, and 690 days, respectively. In Figures S2–S13: *p* indicates the allometric growth level. *p* < 0.05: allometric; *p* ≥ 0.05: isometric. ‘T’ denotes the growth type, with ‘A’ denoting allometric relationships and ‘I’ denoting isometric relationships.

## References

[1] Cao R., Wang H., Zhu Y., Yi M. (2025). The synergistic effect of species diversity and functional diversity in subtropical naturally regenerating forests promotes environmental adaptability of biomass accumulation. Trees For. People.

[2] Doležal J., Chondol T., Chlumská Z., Altman J., Čapková K., Dvorský M., Fibich P., Korznikov K.A., Ruka A.T., Kopecký M. (2024). Contrasting biomass allocations explain adaptations to cold and drought in the world’s highest-growing angiosperms. Ann. Bot..

[3] Geudens G., Staelens J., Kint V., Goris R., Lust N. (2004). Allometric Biomass Equations for Scots Pine (*Pinus sylvestris* L.) Seedlings during the First Years of Establishment in Dense Natural Regeneration. Ann. For. Sci..

[4] Grime J.P. (1977). Evidence for the existence of three primary strategies in plants and its relevance to ecological and evolutionary theory. Am. Nat..

[5] He J., Zhang Y., Xu Z., Shi R., Yang Y., Han D. (2026). Analysis of Allometric Relations of *Picea schrenkiana* in Different Development Stages in the Western Tianshan Mountains of China. Forests.

[6] Huang X., Li S., Su J. (2020). Selective logging enhances ecosystem multifunctionality via increase of functional diversity in a Pinus yunnanensis forest in Southwest China. For. Ecosyst..

[7] Huanca-Nunez N., Chazdon R.L., Russo S.E. (2024). Trait-Mediated Variation in Seedling Performance in Costa Rican Successional Forests: Comparing Above-Ground, Below-Ground, and Allocation-Based Traits. Plants.

[8] Kleyer M., Minden V. (2015). Why functional ecology should consider all plant organs: An allocation-based perspective. Basic Appl. Ecol..

[9] Li N., Zhang X., Cai N., Xu Y. (2026). Grade-specific biomass allocation and allometric growth strategies in *Pinus yunnanensis* seedlings. BMC Plant Biol..

[10] Li S., Wei X., Lin W., Li P. (2026). A novel light use efficiency model incorporating stand age to improve monitoring of mangrove productivity and biomass accumulation. Front. For. Glob. Change.

[11] Lu Z., Wang Q., Yang Z., Chen L., Cai N., Xu Y. (2024). Endogenous Phytohormone and Transcriptome Analysis Provided Insights into Seedling Height Growth of *Pinus yunnanensis*. Forests.

[12] Matías L., Pérez-Ramos I.M., Gómez-Aparicio L. (2019). Are northern-edge populations of cork oak more sensitive to drought than those of the southern edge?. Environ. Exp. Bot..

[13] Niklas K.J., Enquist B.J. (2002). Canonical rules for plant organ biomass partitioning and annual allocation. Am. J. Bot..

[14] Poorter H., Nagel O. (2000). The role of biomass allocation in the growth response of plants to different levels of light, CO_2_, nutrients and water: A quantitative review. Aust. J. Plant Physiol..

[15] Poorter H., Niklas K.J., Reich P.B., Oleksyn J., Poot P., Mommer L. (2012). Biomass allocation to leaves, stems and roots: Meta-analyses of interspecific variation and environmental control. New Phytol..

[16] Bin C. (2004). Discussion on quality evaluation and control method of forestry seedling. Sichuan For. Sci. Technol..

[17] Jeon J., Lim H., Lee K., Noh E.W., Lee I.H., Lee W.Y., Koo Y.B., Jang K. (2025). Evaluating Growth and Stability of Nine Poplar Clones for Riparian Afforestation. Plants.

[18] Gazal R.M., Blanche C.A., Carandang W.M. (2004). Root growth potential and seedling morphological attributes of narra (Pterocarpus indicus Willd.) transplants. For. Ecol. Manag..

[19] Blair R., Cech F. (1974). Morphological seedling grades compared after thirteen growing seasons. Tree Plants’ Notes.

[20] Qi Y., Wei W., Chen C., Chen L. (2019). Plant root-shoot biomass allocation over diverse biomes: A global synthesis. Glob. Ecol. Conserv..

[21] Wang Q., He B., Lu Z., Yang Z., Zheng C., Wang Y., Cai N., Xu Y., Jin J., Wang D. (2024). Variation of Growth Traits and Allometric Growth of Seedlings of Pinus yunnanensis from Different Provenances. J. Southwest For. Univ..

[22] Pu M., Yang S., Chen A., Deng Z. (2025). Research on Forest Carbon Sequestration and Its Economic Valuation: A Case Study of the Zixi Mountain Nature Reserve, Chuxiong Prefecture. Plants.

[23] Schurr U., Walter A., Rascher U. (2006). Functional dynamics of plant growth and photosynthesis–from steady-state to dynamics–from homogeneity to heterogeneity. Plant Cell Environ..

[24] Kenefic L. (2002). Influence of age on growth efficiency of *Tsuga canadensis* and *Picea rubens* trees in mixed-species, multiaged northern conifer stands. Can. J. For. Res..

[25] Liu Z., Gao C., Li J., Miao Y., Cui K. (2022). Phenotypic Diversity Analysis and Superior Family Selection of Industrial Raw Material Forest Species-*Pinus yunnanensis* Franch. Forests.

[26] Singh H., Kumar N., Kumar A. (2024). Enhancing Resource Use Efficiency in Crops Through Plant Functional Traits.

[27] South D.B., Boyer J.N., Bosch L. (1985). Survival and growth of loblolly pine as influenced by seedling grade: 13-year results. South. J. Appl. For..

[28] Sun Y., Zhao W., Xu C., Xu Y., El-Kassaby Y.A., De La Torre A.R., Mao J. (2020). Genetic variation related to high elevation adaptation revealed by common garden experiments in *Pinus yunnanensis*. Front. Genet..

[29] Wang P., Zhou C., Yang B., Li J., Xu Y., Cai N. (2025). Allometric Growth of Annual *Pinus yunnanensis* After Decapitation Under Different Shading Levels. Plants.

[30] Tang G., Wang Y., Lu Z., Cheng S., Hu Z., Chen S., Chen L., Tang J., Xu Y., Cai N. (2024). Effects of Combined Nitrogen–Phosphorus on Biomass Accumulation, Allocation, and Allometric Growth Relationships in *Pinus yunnanensis* Seedlings after Top Pruning. Plants.

[31] Wang P., Lu Z., Xu Y., Cai N. (2026). Allometric Growth Patterns and Phenotypic Plasticity Indices of Different Grades of Annual Pinus yunnanensis Franch. Seedlings at Different Growth Stages. Biology.

[32] Montagnoli A., Terzaghi M., Fulgaro N., Stoew B., Wipenmyr J., Ilver D., Rusu C., Scippa G.S., Chiatante D. (2016). Non-destructive Phenotypic Analysis of Early Stage Tree Seedling Growth Using an Automated Stereovision Imaging Method. Front. Plant Sci..

[33] Wang Q., Yan T., Wang Y., Li Y., Wang D., Chen S., Chen L., Tang J., Cai N., Xu Y. (2022). Gradei-fication and allometric growth of annual *Pinus yunnanensis* seedlings from different provenances. Acta Cent. South Univ. For. Technol..

[34] (2011). Fast-Growing and High-Yielding Plantation of *Pinus yunnanensis*.

[35] Deng X., Xiao W., Shi Z., Zeng L., Lei L. (2020). Combined Effects of Drought and Shading on Growth and Non-Structural Carbohydrates in *Pinus massoniana* Lamb. Seedlings. Forests.

[36] Deng X., Chen X., Li T., Zhang H., Bao Y., Yang J., Zheng L., Lan P., Wu J. (2025). The Effects of Drought Stress Intensity and Duration on the Dynamics of Nonstructural Carbohydrates in *Pinus yunnanensis* Seedlings. Plants.

[37] Enquist B.J., Niklas K.J. (2002). Global Allocation Rules for Patterns of Biomass Partitioning in Seed Plants. Science.

[38] Falster D.S., Warton D.I., Wright I.J. (2020). User’s Guide to SMATR: Standardised Major Axis Tests & Routines Version 2.0, Copyright 2006. http://www.bio.mq.edu.au/ecology/SMATR/.

[39] Li Y., Chen S., Sun J., Wang J., Xu Y., Cai N. (2020). The Relationships Between Classification and Biomass Allocation of 2 Years Old *Pinus yunnanensis* Seedlings. J. Southwest For. Univ..

[40] Pan Z., Lu Z., Li S., Liao J., Zhou C., Chen L., Chen S., Cai N., Wang D., Xu Y. (2025). Seasonal Variation in Root Morphological Traits and Non-Structural Carbohydrates of *Pinus yunnanensis* Seedlings Across Different Seedling Orders. Plants.

[41] Tang C.Q., Shen L., Han P., Huang D., Li S., Li Y., Song K., Zhang Z., Yin L., Yin R. (2020). Forest characteristics, population structure and growth trends of *Pinus yunnanensis* in Tianchi National Nature Reserve of Yunnan, southwestern China. Veg. Classif. Surv..

[42] Wang L., Huang X., Su J. (2022). Tree Species Diversity and Stand Attributes Differently Influence the Ecosystem Functions of Pinus yunnanensis Secondary Forests under the Climate Context. Sustainability.

[43] Weiner J. (2004). Allocation, plasticity and allometry in plants. Perspect. Plant Ecol. Evol. Syst..

[44] West P.W. (2024). A review of the growth behaviour of stands and trees in even-aged, monospecific forest. Ann. For. Sci..

[45] Xu J., Li X., Chen S., Chen L., Tang J., Chen P., Cai N., Xu Y. (2025). Integrative analyses of morpho-physiological, biochemical, and transcriptomic reveal the seedling growth response of *Pinus yunnanensis* to nitrogen and phosphorus fertilization. Front. Plant Sci..

[46] Hou J., Yan L., Zhao G., Du T., Shao S. (2015). Relationship Between Seedling Grade of Glycyrrhiza uralensis and Plant Biomass Accumulation, Yield and Quality of Product. Zhong Yao Cai = Zhongyaocai = J. Chin. Med. Mater..

[47] Li R., Wang Q., Li S., Chen L., Cai N., Xu Y. (2025). Study on the growth characteristics of *Pinus yunnanensis* seedlings based on hormonal regulation and chlorophyll metabolism. Sci. Rep..

[48] Ye H.-Y., Yan Z.-B., Ma S.-H., Yang X.-M., Yang C., Zhang D.-H., Zhu J.-L., Ji C.-J., Fang J.-Y. (2025). Mediation of stem photosynthesis in biomass accumulation and allocation in Rosa chinensis. J. Plant Ecol..

[49] Wang F., Yang Y., Wang J., Liu Y., Wang H., Song Y., Lu Z., Yang Y. (2024). Biomass Allocation and Allometry in *Juglans mandshurica* Seedlings from Different Geographical Provenances in China. Forests.

[50] Zhang F.F., Li X.Q., Wu P.P., Zhong Q.L., Hu D.D., Cheng D.L. (2024). Biomass allocation characteristics and model construction of seedlings of six broad-leaved tree species. J. For. Environ..

[51] Younginger B.S., Sirová D., Cruzan M.B., Ballhorn D.J. (2017). Is biomass a reliable estimate of plant fitness?. Appl. Plant Sci..

[52] Zhu Y., Wang X., Wang D., Tang J., Chen S., Cai N., Xu Y. (2021). Analysis on Growth and Allometric Growth of *Pinus yunnanensis* Seedlings with Different Stem Types. J. Yunnan Agric. Univ. (Nat. Sci.).

[53] Iida Y., Swenson N.G. (2020). Towards linking species traits to demography and assembly in diverse tree communities: Revisiting the importance of size and allocation. Ecol. Res..

